# Bibliographical

**Published:** 1893-10

**Authors:** 


### Bibliographical.

The New Illustrated Dictionary of Medicine, Biology and Collateral Sciences, by Dr. George M. Gould, of Philadelphia.

This volume, following so soon the issue of the dictionary of Dr. Gould in 1890, is a strong evidence of the appreciation of the work he has done, and a clear indication of the faith of the medical profession in what he can do in the line of lexicography.

We have just received some advance sheets of the new work, and if these may be taken as an indication of the character of the whole work it is not too much to say that it will be superior to anything of the kind ever brought to the attention of the profession. It is unique in its epitomization of old and new, in which it is to contain a far larger number of words than any other one volume lexicon. It is a new book; not a revision of the older volume.

We shall await with interest and anxious expectation the issue of the work, when it will receive a more extended notice.

It is published by P. Blakiston, Son & Co., Philadelphia.

